# ROC curve analyses of eyewitness identification decisions: An analysis of the recent debate

**DOI:** 10.1186/s41235-016-0006-7

**Published:** 2016-09-22

**Authors:** Caren M. Rotello, Tina Chen

**Affiliations:** Department of Psychological & Brain Sciences, University of Massachusetts, 135 Hicks Way, Amherst, MA 01003-9271 USA

**Keywords:** Eyewitness identifications, Lineups, Showups, Receiver operating characteristic curve (ROC), Decision accuracy, Diagnosticity

## Abstract

How should the accuracy of eyewitness identification decisions be measured, so that best practices for identification can be determined? This fundamental question is under intense debate. One side advocates for continued use of a traditional measure of identification accuracy, known as the *diagnosticity ratio*, whereas the other side argues that receiver operating characteristic curves (ROCs) should be used instead because diagnosticity is confounded with response bias. Diagnosticity proponents have offered several criticisms of ROCs, which we show are either false or irrelevant to the assessment of eyewitness accuracy. We also show that, like diagnosticity, Bayesian measures of identification accuracy confound response bias with witnesses’ ability to discriminate guilty from innocent suspects. ROCs are an essential tool for distinguishing memory-based processes from decisional aspects of a response; simulations of different possible identification tasks and response strategies show that they offer important constraints on theory development.

## Significance

Eyewitness identifications can provide compelling evidence in criminal cases. For decades, researchers have explored the factors that influence the accuracy of those memory judgments, including consideration of both *system variables* that are controlled by the legal system (e.g., identification procedures and instructions) and *estimator variables* that are intrinsic to the witness’s experience of the crime (e.g., viewing distance, stress). The accuracy of the identification itself has almost always been summarized with a measure known as *diagnosticity*, which is simply a ratio of correct and incorrect identification probabilities. Crucially, recent research has demonstrated that diagnosticity is confounded with witnesses’ willingness to identify a suspect, raising questions about the validity of prior conclusions about the effects of system and estimator variables. Those conclusions rest on the assumption that manipulations of these variables affect only the accuracy of the identification, but the diagnosticity measure cannot distinguish between an effect on accuracy and an effect on identification willingness to identify. More appropriate measures of eyewitness identification accuracy can be obtained from receiver operating characteristic curves (ROCs) based on confidence ratings. Although ROCs have a long and productive history in many domains of psychological research (including recognition memory), their application to eyewitness identification decisions has been challenged. In this paper, we correct some of the conceptual errors in the literature on identification ROCs, and demonstrate their value for both understanding the factors that influence eyewitness identification accuracy and constraining theoretical development.

## Measuring eyewitness information accuracy

How should the accuracy of eyewitness identification decisions be measured so that best practices for identification can be determined? This fundamental question is under intense debate. One side of the argument (e.g., Wells, Yang, & Smalarz, 2015a) is that the probative measure that has been used for decades, namely the ratio of correct identifications of guilty suspects to false identifications of innocent suspects (i.e., hit rate/false alarm rate = *H*/*F*; Wells & Lindsay, 1980, Equation 6), provides the best measure of eyewitness accuracy. This ratio is known as the *diagnosticity ratio*, or simply as *diagnosticity*. The other side of the argument is that diagnosticity is confounded with witnesses’ tendency to identify someone; that is, to “choose” from the lineup (e.g., Wixted & Mickes, 2012). To the extent that this response bias varies across identification procedures, diagnosticity will also vary, even if witnesses are equally able to discriminate guilty from innocent suspects. For this reason, basing a preference for a particular identification procedure on an observed difference in diagnosticity ratios (e.g., Steblay, Dysart, & Wells, 2011) is a deeply flawed approach.

An alternative to the diagnosticity ratio is to use the confidence ratings that are routinely collected with identification decisions to construct ROCs (e.g., Mickes, Flowe, & Wixted, 2012; Wixted & Mickes, 2012). In the context of eyewitness identifications, ROCs usually plot the probability of a correct identification of a guilty suspect (*H*) against the probability of a false identification of an innocent suspect (*F*) at each confidence level or response bias. By definition, the empirical points on any given ROC differ only in terms of response bias, whereas ROC that fall higher in the space reflect greater ability to discriminate guilty from innocent suspects because the hit rate is higher for any particular false alarm rate. Levi (2016, p. 45) claimed that “[an eyewitness] ROC fails to produce a discriminability measure,” and Wells et al. (2015a, p. 118) wrote that “it is not clear that the ROC approach is properly controlling for response bias or that it measures discriminability.” Both claims are wrong. In fact, that is exactly what ROCs do.

A good quantitative measure of discrimination accuracy that is independent of response bias is the area under the ROC (AUC) (Green & Swets, 1966; Macmillan & Creelman, 2005; Pollack & Hsieh, 1969). For eyewitness identification tasks, a partial area under the curve (pAUC) is typically reported because the false alarm rate is naturally limited by the number of photographs in the lineup, resulting in a curve that does not extend across the entire *x*-axis (e.g., Mickes et al., 2012). The AUC and pAUC measure the ability of witnesses to discriminate between the two classes of stimuli that are summarized on the *x*- and *y*-axes, independently of response bias. For example, if identifications of guilty suspects are plotted on the *y*-axis and identifications of innocent suspects on the *x*-axis, then the area under the curve reflects the ability of witnesses to distinguish between guilty and innocent suspects. This measure of discrimination is crucial for determining which identification procedure results in the highest-accuracy classifications of suspects as either guilty or innocent. On the other hand, if the *x*-axis includes *any* positive identification from a target absent (TA) lineup (including filler identifications), as Wells and colleagues proposed (Wells, Smalarz, & Smith, 2015b; Wells, Smith, & Smalarz, 2015c), then the AUC reflects the ability of witnesses to discriminate guilty suspects (in a target present [TP] lineup) from everybody in the TA lineup. We do not see the relevance of this measure for policy makers, the legal system, or eyewitness researchers. However, observed differences between this AUC (which measures discrimination of a guilty suspect from all the fillers in the TA lineup) and the AUC derived from an ROC that plots identifications of guilty and innocent suspects have been used as a criticism of eyewitness ROCs. We will elaborate this point shortly.

The best measure of eyewitnesses’ ability to discriminate guilty from innocent suspects is one that does not conflate decision accuracy and response bias. Factors such as the base rate of lineups that contain a guilty suspect, or the costs and benefits of correct and erroneous identification decisions (i.e., falsely identifying an innocent suspect; failing to identify a guilty suspect), contribute to determining the optimal decision criterion or response bias (i.e., the criterion location that maximizes the expected value of the decision; Macmillan & Creelman, 2005, Equation 2.8). However, these factors do not have any bearing on an appropriate measure of discrimination. The AUC is one such measure.

ROC analyses of eyewitness identifications have been roundly criticized (e.g., Lampinen, 2016; Wells et al., 2015b). Indeed, Levi (2016) wrote that “researchers are warned against using ROC[s] when conducting lineup research” (p. 42). One primary argument against ROCs is that witness responses fall into three categories (identify a suspect, identify a filler, or reject the lineup), but ROCs require the data to be collapsed into two categories (positive and negative responses; Wells et al., 2015b). Because suspect identifications are of primary interest to law enforcement, virtually all reported ROCs have combined filler identifications and lineup rejections into the set of negative responses, leaving only suspect identifications as positive responses. This treatment of the data is exactly the same as is used in the calculation of the diagnosticity ratio (Wells & Lindsay, 1980, Equation 6), so the fact that data are collapsed is not a criticism of ROCs per se.^1^ Indeed, as we suggested earlier, we believe that ROCs based only on suspect identifications are precisely the data needed to assess witnesses’ ability to discriminate guilty from innocent suspects independently of their willingness to identify someone.

An intimately related argument is that collapsing filler identifications with lineup rejections ignores “filler siphoning.” Filler siphoning is the observation that erroneous identifications in a showup procedure (or a strongly biased lineup) all implicate the innocent suspect, whereas in a fair lineup procedure, those errors are distributed across the suspect and the fillers (Wells et al., 2015b). In other words, false identifications of the innocent suspect are lower in a fair lineup because there are other photographs that the witness might plausibly choose; correct identifications of the guilty suspect also tend to be lower, for the same reason.^2^ Despite claims made by Wells et al. (2015c), the existence of filler siphoning has no bearing on the appropriateness of ROCs for the analysis of eyewitness identifications (see also Wixted & Mickes, 2015a, 2015b). As we described earlier, including positive responses to known innocent fillers on the *x*-axis of the ROC changes what the ROC measures, as we elaborate next (see Wixted & Mickes, 2015b for a detailed quantitative demonstration of this point).

Using Wetmore et al. (2015) data, Wells et al. (2015c) showed that the AUC is greater for fair than for biased lineups when the ROC includes only positive responses to the guilty and innocent suspects; these ROCs reflect witnesses’ ability to discriminate guilty from innocent suspects in the two tasks, indicating that higher-accuracy decisions result from fair lineups. Wells et al. (2015c) also showed that the AUC is lower for fair than biased lineups when filler identifications are included in the false alarm rate, producing an ROC that shows how well witnesses can discriminate the guilty suspect in a TP lineup from everybody in the TA lineup (a measure of questionable value). The reason the AUC is higher for the biased lineups in this analysis is that some of the fillers did not look like the perpetrator in Wetmore et al.’s experiment (Tredoux’s, 1998; E′ averaged 2.74). In contrast, in the fair lineups, the fillers were more plausible candidates for identification (E′ averaged 4.12), meaning that it was simply easier to discriminate the guilty suspect in the TP lineup from all of the TA fillers in the biased case than in the fair case. But as has been mentioned already, the fact that the relative AUC changes depending on whether positive identifications of known innocent fillers are included on the *x*-axis of the ROC is irrelevant to the question of interest: Which identification procedure best distinguishes guilty from innocent suspects? As Wells et al. (2015c) clearly showed, the AUC for that comparison is larger for fair lineups.

One recent paper, by Lampinen (2016), has the potential to play an important role in the debate about diagnosticity and the AUC, because it appears to offer a sophisticated modeling approach and has been interpreted as providing “strong additional evidence that ROC analyses on lineups are not measures of discriminability” (Wells et al., 2015b, p. 316). Lampinen’s analyses, if correct, would present a challenge to ROC analyses of eyewitness identifications. For this reason, we respond to his criticisms in detail in the next two sections, showing them to be faulty. In the third section, we evaluate Bayesian measures of eyewitness performance and show that they reflect a complex mixture of true discriminability of guilty from innocent suspects, response bias, and the probability that a guilty suspect is presented to the witness. Finally, we conclude by considering the potential value of identification ROCs for development of better theories of eyewitness memory and decisions.

## Lampinen’s ROC criticisms

Using simulated data, Lampinen (2016) argued that ROCs derived from lineup and showup identification procedures produce different estimates of witnesses’ ability to discriminate guilty from innocent suspects even if their true level of discrimination accuracy does not vary. This point, if correct, would raise questions about ROC-based comparisons of the accuracy of lineup and showup identifications (Gronlund et al., 2012; Mickes, 2015; Wetmore et al., 2015). However, we will show that Lampinen reached an erroneous conclusion because he simulated data using relatively liberal criterion locations and inappropriately applied signal detection equations from a different decision task. Lampinen’s second criticism of ROCs is that they encourage researchers to compare discrimination accuracy at different levels of witness confidence, which “is not a reasonable or scientifically valid way to compare two conditions” (Lampinen, 2016, p. 28). As we will argue, this claim fails to recognize the most valuable contribution of ROCs, which is exactly that they eliminate the need to worry about witness confidence because the same decision accuracy (*d′*) is reflected at *every* confidence level.1. In contrast to Lampinen’s (2016) claim, ROCs provide the best measures of underlying discrimination performance and may be compared across lineups of different lengths (including showups).

Lampinen (2016) argued that witnesses’ ability to distinguish guilty from innocent suspects appeared to be different for showup and lineup procedures that involve ROCs, so “pAUC analyses do not provide a valid way of comparing identification procedures” (Lampinen, 2016, p. 26). As evidence, he offered a series of simulations in which true discrimination accuracy (*d′*) was equated for two different identification tasks: showups and six-photograph lineup identifications. In both cases, Lampinen assumed an underlying representation based on signal detection theory (Macmillan & Creelman, 2005). Specifically, memory strength values were sampled from Gaussian strength distributions with a mean of 0 and a standard deviation of 1 for fillers, and a mean of *d′* (set at 0.5, 1, 1.5, or 2)^3^ and a standard deviation of 1 (for equal variance simulations) or 1.2 (for unequal variance) for guilty suspects.

To simulate the showup procedure, Lampinen randomly selected a single value from either the guilty suspect distribution, to represent a TP showup, or the foil distribution, to represent a TA showup. The sampled strength was compared with a fixed set of criterion values (0.18, 0.23, 0.27, 0.39, 0.67, 1.15, and 1.53) to assign a confidence level for the response. For example, a sampled strength of 1.0 would be assigned a confidence level of 6 because it falls in the interval between the fifth and sixth criterion locations. Any sampled strength greater than 0.18, the lowest criterion, was assumed to result in a positive identification.

Simulation of the six-photograph lineup procedure was similar, except that each simulated lineup involved six sampled strengths, either one from the guilty suspect distribution and five from the filler distribution (for a TP lineup) or all six from the filler distribution (for a TA lineup). In either case, the highest sampled strength determined the confidence rating. If that strength was from the guilty suspect distribution, then a “hit” resulted. If the lineup included only fillers, then any strength above the lowest criterion (0.18) was treated as a false alarm. Because there are six opportunities for a filler to exceed the criterion in a TA lineup, the resulting response rates were divided by 6.

Lampinen (2016) showed that the simulated ROC for the showup procedure fell above the simulated ROC for the lineup procedure for each of the true *d′* values he considered. Because the AUC is a measure of subjects’ ability to discriminate between two types of stimuli (i.e., guilty and innocent suspects; Green & Swets, 1966; Macmillan & Creelman, 2005), he concluded that the estimated accuracy was higher for the showup method. As a consequence, his simulations seem to suggest that the AUC fails to provide a good measure of eyewitnesses’ ability to discriminate guilty from innocent suspects. We will show, using our own simulations, that ROCs based on a choice from among a larger set of options (i.e., six-person lineup rather than three-person one) do have lower hit and false alarm rates at every criterion location, though for reasons quite different from those offered by Lampinen. However, the difference in the AUCs (which Lampinen did not report) is inconsequential compared with the difference in AUCs when true discrimination accuracy (*d′*) actually varies.

We performed simulations similar to Lampinen’s so that the AUCs could be calculated. To extend and generalize his analyses, we varied the criterion locations continuously for 10,000 different simulated trials of each type, thus mapping out the full theoretical ROC. By comparison, Lampinen assumed a particular set of fixed criterion locations, which necessarily provides only a snapshot of the ROC. In addition, we assumed several different lineup sizes (2, 3, or 6). Although lineups with only two or three photographs are not used in standard police procedures, their inclusion in our simulations does provide some insight into the relationship between lineup size and form of the ROC.

**Fig. 1:**
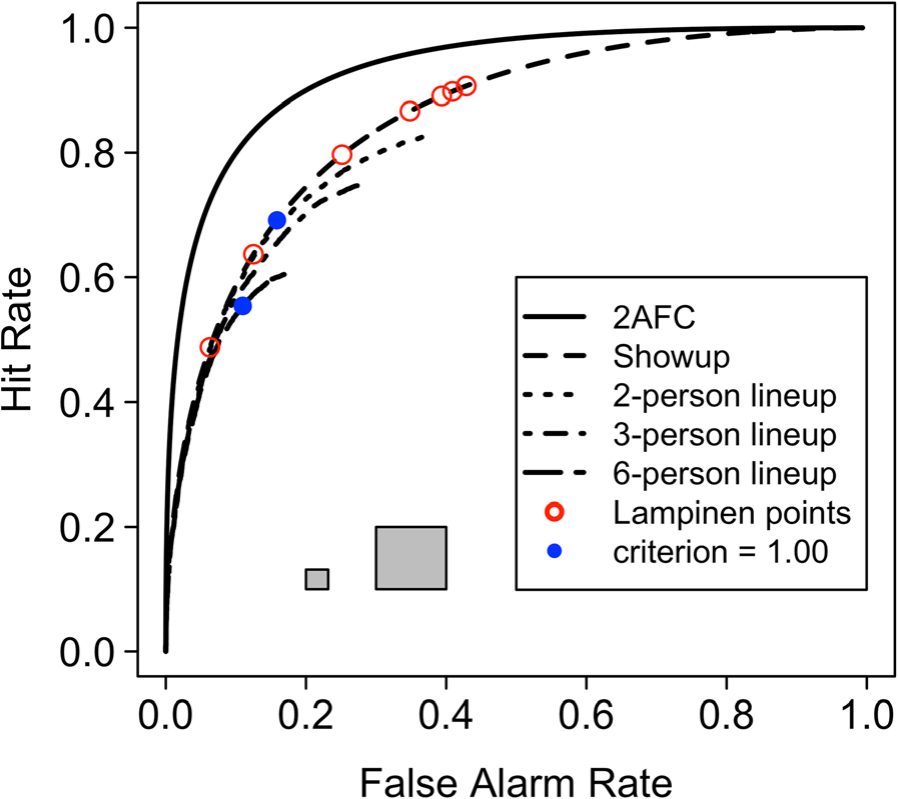
Simulated receiver operating characteristic curves (ROCs) assuming true *d′* = 1.5 and equal variance Gaussian distributions for five different recognition memory tasks. The *larger gray square* shows an area under the ROC (AUC) = 0.01; the *smaller gray square* shows an AUC = 0.001. Points marked with a *red open circle* are the operating points simulated by Lampinen (2016); points marked with a *blue filled circle* are the operating points that result from a decision criterion of 1.00 in the showup and six-person lineup tasks. *2AFC* = two-alternative forced choice

Our simulated showup and lineup ROCs are shown as the set of ROCs in Fig. 1 that are represented with dashed functions. These curves were generated using one of the combinations of parameter values from Lampinen’s study, namely a true *d′* of 1.5 and equal variance distributions. (Other values of true *d′* yielded similar results.) Notice that these four ROCs are visually indistinguishable at the lowest false alarm rates (up to about 0.10) that reflect the highest confidence levels. This part of the ROC was not shown in any of Lampinen’s simulations, because the most conservative criterion he selected was relatively liberally placed and thus produced high hit and false alarm rates overall. The operating points in Lampinen’s simulations are marked with *red open circles* on the showup ROC in Fig. 1.

The highest-confidence identification decisions are those that are most valuable to the legal system because they carry the greatest probative value (Wixted, Mickes, Dunn, Clark, & Wells, 2016). High-confidence identifications are much more likely to be correct than low-confidence identifications (e.g., Carlson, Dias, Weatherford, & Carlson, in press; Juslin, Olsson, & Winman, 1996; Mickes, 2015; Palmer, Brewer, Weber, & Nagesh, 2013; Wixted et al., 2016), and jurors are more likely to believe confident witnesses (e.g., Cutler, Penrod, & Stuve, 1988). Thus, the highest-confidence and most important region of the showup and six-person lineup ROCs are visually indistinguishable when true accuracy (*d′*) is equated. Lampinen’s selection of particular, relatively liberal decision criteria obscured this similarity.

At lower levels of confidence, or more liberal response biases, it becomes apparent that the number of response options affects both the hit and false alarm rates. The false alarm rate is limited for the obvious reason that selection of any of the fillers in a TA lineup is an error; chance response rates are properly limited to 1/*N*, where *N* is the lineup size. The basis for the decrease in the hit rate with increasing lineup size is perhaps less obvious. Because witnesses select the photograph that is most familiar (assuming it exceeds some criterion), the greater the number of fillers in a TP lineup, the more likely it is that one of them will have greater familiarity than the guilty suspect just by chance. In that case, the witness would choose a filler instead of the guilty suspect, thus reducing the hit rate. A consequence of these two effects is that the very same evidence values in memory, used in conjunction with identical criterion locations, can nonetheless result in hit and false alarm rates that are reduced as the number of photographs in the lineup increases. For example, the *blue circles* in Fig. 1 show the hit and false alarm rates that result when witnesses have a true *d′* value of 1.5 and use a decision criterion of 1.00 for both showup and six-person lineup decisions: Their decisions appear more conservative in the lineup task simply as a consequence of the number of photographs presented (both memory and the decision process are identical).

To estimate the area under the curve for each of the ROCs shown in Fig. 1, we used the R package pROC (Xavier et al., 2011). Because the maximum false alarm rate is limited by the lineup size, we actually estimated the pAUC for the showup and six-person lineup ROCs (those compared visually by Lampinen) using a false alarm rate range of 0 to either 0.10 (only the highest-confidence responses) or 0–0.16 (essentially the full ROC for the six-person lineup). To obtain confidence intervals on these area estimates, we repeated this process using 2000 bootstrapped samples for each identification procedure and true accuracy level (*d′*), selecting the pAUCs at the 2.5 and 97.5 percentiles as the lower and upper bounds of the 95 % confidence intervals.

**Fig. 2:**
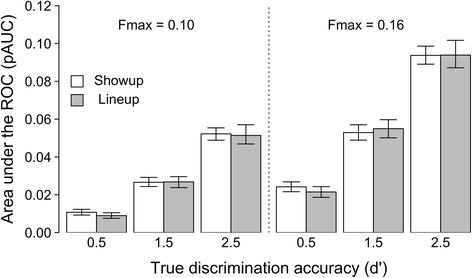
Simulated partial area under the receiver operating characteristic curve (pAUC) for showup and six-person lineup identifications assuming three different levels of true discrimination accuracy (*d′*). *Left panel*: *F*_max_ = 0.10; *right panel*: *F*_max_ = 0.16. Error bars are 95 % confidence intervals estimated with 2000 bootstrapped samples

Figure 2 shows that the difference in pAUCs for showups and six-person lineups is trivial when true accuracy (*d′*) is equated.^4^ The differences typically appear in the fourth decimal place (i.e., less than the size of the *smaller gray square* in Fig. 1). In contrast, when true accuracy (*d′*) varies, the pAUC for both showup and six-person lineup identifications capture those changes quite readily. Figure 2 shows that with a true accuracy difference of 1.0 *d′* units, pAUC changes are obvious for both showup and lineup decisions (i.e., approximately twice the size of the *larger gray square* in Fig. 1). Thus, contrary to Lampinen’s conclusion, we observe that the AUCs obtained from showup and six-person lineup identifications may be safely compared empirically: They yield indistinguishable estimates of discrimination accuracy when true accuracy (*d′*) is held constant and change appropriately (and quite similarly) when true accuracy varies.^5^

Of course, these simulations *assume* witnesses are equally good at discriminating guilty from innocent suspects in showup and lineup tasks. On one hand, they show that, theoretically, *if* discrimination accuracy (*d′*) is the same, the ROCs are highly unlikely to suggest that either of the procedures results in higher-accuracy decisions than the other. On the other hand, the simulations also show that real differences in discrimination accuracy (*d′*) *can* be detected with ROCs, and to the same degree for both showups and lineups. Whether accuracy is actually the same in these two identification procedures is an empirical question, not a theoretical one. Empirical comparisons of ROCs for showup and lineup identifications have consistently revealed that showups result in significantly lower decision accuracy than lineup procedures, as measured with pAUC (Gronlund et al., 2012; Mickes, 2015; Wetmore et al., 2015). Our simulations suggest that the most appropriate conclusion to draw from these studies is that showup identification accuracy is inferior to lineup identification accuracy. (See Wixted and Mickes [2014] for a possible theoretical explanation of that difference.)

The other published ROC comparisons of eyewitness identifications are safe from Lampinen’s criticism as well. Lampinen’s basic claim was that varying the length of the lineup (from six to one) affected estimated but not true witness accuracy, but the primary application of ROCs to eyewitness identification decisions has been to compare sequential and simultaneous presentation of the same lineup photographs. The consistent finding, that pAUC for simultaneous lineups is equal to or greater than for sequential procedures (Carlson & Carlson, 2014; Dobolyi & Dodson, 2013; Gronlund et al., 2012; Mickes et al., 2012), is not in any way challenged by Lampinen’s arguments, because the same lineup length (indeed, the same lineup) was used for both procedures. Thus, the ROC data indicate that, if anything, there is a simultaneous superiority effect; we will revisit this issue in the final section of this paper, where we discuss the implications of ROC data for theoretical developments in eyewitness identifications.

Finally, note that the “proof” offered by Lampinen (2016, Appendix) of the relationship between estimated discrimination accuracy from lineups and showups is irrelevant because that math applies for a different task, namely a two-alternative forced choice (2AFC) task. In a 2AFC task, participants are shown a target and a lure and must choose the target. The decision is usually modeled as one of taking a difference between the two (independent) memory strengths, and thus the distribution of interest becomes *N*(*d′*, √[1^2^ + *s* ^2^]), where *s* is the standard deviation of the guilty suspect distribution.^6^ The resulting ROC, again for a true *d′* of 1.5 and *s* = 1, is shown as the solid curve in Fig. 1. This is not the ROC simulated by Lampinen, nor is it the same ROC that occurs for lineups of size 2 (see Fig. 1). Participants in an eyewitness identification task have the option of rejecting the lineup entirely, which may change the underlying task from one of comparison (as in 2AFC) to one in which the subject must simply identify the strongest item that exceeds a minimum criterion (DeCarlo, 2013). Comparisons of ROCs from different tasks should involve careful consideration of the decision processes and memory evidence involved, as we will show in the final section of this paper.

To summarize, Lampinen’s first criticism misses the mark in several important ways. It is irrelevant to the comparison of sequential and simultaneous lineups that has dominated the eyewitness ROC literature, and it reaches the unfounded conclusion that estimated accuracy (pAUC) differs systematically for showups and lineups. Our analyses demonstrate that the two paradigms yield essentially identical estimates of performance when the true accuracy level (*d′*) is equated. Thus, Lampinen’s argument does not change either the empirical conclusion that simultaneous lineups yield equal or greater AUCs than sequential lineups (Carlson & Carlson, 2014; Dobolyi & Dodson, 2013; Gronlund et al., 2012; Mickes et al., 2012), or that showups yield lower-accuracy identifications than lineups of either type (Gronlund et al., 2012; Mickes, 2015; Wetmore et al., 2015). Our comparison of showup and six-person lineup ROCs confirms that these ROC analyses provide accurate information about relative performance.2. Lampinen (2016) claims ROCs invite inappropriate comparison of accuracy at different levels of response bias. In truth, ROCs separate bias from discrimination accuracy.

Lampinen’s (2016) second major claim is that ROCs invite comparison of memory accuracy across different levels of confidence. As an example, he selects a particular false alarm rate, say, 0.167 (see Lampinen, 2016, Fig. 6), and then observes that the hit rates vary for different simulated identification procedures, suggesting that different estimates of accuracy (say, *d′*) would be observed for lineup and showup identifications. Finally, Lampinen notes that if response bias differs across identification procedures, then the operating points being compared may reflect different degrees of witness confidence. Conversely, Levi (2016) worries that witnesses given different identification procedures may respond with the same confidence level despite having different discrimination accuracy (*d′*).

These criticisms completely miss the value of ROCs, namely that each ROC reflects the *same* accuracy (*d′*) at every point. Because of this property of ROCs, one can readily see both accuracy and response bias effects simultaneously: Curves higher in the space reflect higher (*d′*) decision accuracy (though possibly not meaningfully so, as our simulations demonstrate), and points toward the lower-left end of the curve reflect more conservative responses. One does not need to compute a single-point measure of accuracy, such as *d′*, at a given false alarm rate to compare accuracy across two conditions. Indeed, one *should not* do so, because *d′* is confounded with response bias whenever the underlying evidence distributions have unequal variance, as is consistently observed in recognition memory judgments (e.g., Ratcliff, Gronlund, & Sheu, 1992). This mistake has led to substantial interpretation errors in a variety of memory experiments (e.g., Dougal & Rotello, 2007; Evans, Rotello, Li, & Rayner, 2009; Verde & Rotello, 2003).

It is important to understand that every “single-point” measure of decision accuracy, be it *d′*, percent correct, diagnosticity, or something else, has an associated theoretical ROC. The theoretical ROC for a given measure simply connects all combinations of hit and false alarm rates that yield the same accuracy value according to that measure, regardless of differences in response bias. However, the various single-point measures of accuracy each predict a different ROC form, which means that researchers who ignore differences in response bias across conditions may easily (and erroneously) conclude that accuracy differs if they select an inappropriate accuracy measure (see Dube, Rotello, & Heit, 2010, and Rotello, Masson, & Verde, 2008, for detailed explanations). For example, for a constant value of diagnosticity, *D* = *H* / *F*, it is easy to see that the theoretical ROC, *H* = *D* × *F*, is a line with an intercept of 0 and a slope equal to the diagnosticity value itself. Rotello, Heit, and Dubé (2015, Fig. 1) plotted several such theoretical ROCs for diagnosticity and compared them with the empirical ROCs reported in a study of eyewitness identifications (Mickes et al., 2012, Experiment 1b). The empirical ROCs were curved, not linear, meaning that different empirical response biases would yield different estimated diagnosticity despite actually representing the same underlying discrimination accuracy (*d′*). For this reason, diagnosticity is not an appropriate measure of eyewitness identification accuracy (nor for any other task of which we are aware; see Swets, 1986a).

**Fig. 3:**
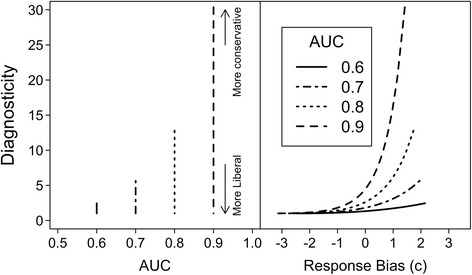
*Left panel*: Diagnosticity (hit rate/false alarm rate) as a function of true area under the receiver operating characteristic curve (AUC = 0.6, 0.7, 0.8, or 0.9). For each AUC level, the false alarm rate varies from 0.01 (more conservative) to 0.99 (more liberal); given each false alarm rate and AUC, the hit rate is determined by *H* = Φ[√2 *z*(AUC) + *z*(*F*)]. *Right panel*: For the same AUC, hit rates, and false alarm rates, diagnosticity is plotted as a function of response bias [measured with *c* = −0.5(*zH* + *zF*)]

When the empirical and theoretical ROCs do not match, as for the empirical identification ROCs and the diagnosticity measure, a different accuracy measure must be selected; otherwise, estimated accuracy and response bias are confounded (Rotello et al., 2008; Swets, 1986b; Wixted & Mickes, 2012). For this reason, Lampinen’s claim that ROCs and probative value measures such as diagnosticity provide redundant information (p. 32; see also Levi, 2016, p. 45) is simply incorrect unless response bias is the measure of interest. As Fig. 3 shows, diagnosticity varies systematically with response bias but is uncorrelated with any particular AUC. Importantly, the greatest variability in diagnosticity estimates occurs for the most conservative response biases (i.e., those with larger positive values of the signal detection measure *c*) that yield the high-confidence responses that are most useful to the legal system (e.g., Wixted et al., 2016).

In summary, ROCs do not “invite” inappropriate comparisons of performance across different response biases. Instead, they make explicit if and how response bias differs across empirical conditions, and they yield a measure of response accuracy (AUC) that is independent of response bias. In contrast, single-point measures, such as *d*′, percent correct, and diagnosticity, both obscure and are almost invariably confounded with differences in response bias.

## Bayesian measures of eyewitness identification

Bayesian measures of eyewitness identification decisions have also been proposed (Wells & Lindsay, 1980) and are argued to offer quite specific quantitative information about the probability that a suspect is guilty. The authors of one recent paper (Wells et al., 2015a) presented an analysis of the relative amount of information gained from an identification decision (either positive or negative) under a range of different conditions, such as whether the lineup was conducted using a sequential or simultaneous procedure or whether the fillers were similar or dissimilar to the suspect. The *information gain* they reported is the difference in the probability that the suspect is actually guilty before and after an identification (or lineup rejection) occurs (i.e., the difference in the prior and posterior probabilities of guilt). The information gain curves they reported vary over the probability that the lineup includes a guilty suspect (i.e., the base rate of TP lineups) and as a function of witness confidence.

What is missing in the discussion of these Bayesian measures is any acknowledgement that the posterior probability of guilt, and consequently the information gain curves, also depends on the underlying ability of witnesses to discriminate guilty from innocent suspects (*d′*) in each task. For example, Wells et al. (2015a) interpreted the lower information gain provided by a suspect identification when the filler photographs are dissimilar rather than similar to the suspect as indicating that the lineup is suggestive of guilt—a biasing effect that would tend to increase both true and false identifications. Of course, it is also possible that the presence of highly similar foils simply makes the memory task harder, decreasing witnesses’ ability to discriminate guilty from innocent suspects. Indeed, Clark’s (2012) meta-analysis suggests that both response bias and discrimination accuracy (*d′*) change as a function of filler similarity. The posterior probability of guilt and information gain plots do not allow us to distinguish between these two interpretations of the data: Both discrimination and response bias contribute to each curve. Similarly, Wells et al. (2015a) noted that greater information gains result from a suspect identification in a sequential rather than simultaneous lineup. Given that sequential lineups result in lower AUCs (e.g., Mickes et al., 2012), this effect is most likely due to the well-documented response bias differences that these two procedures induce (e.g., Clark, 2012).

**Fig. 4:**
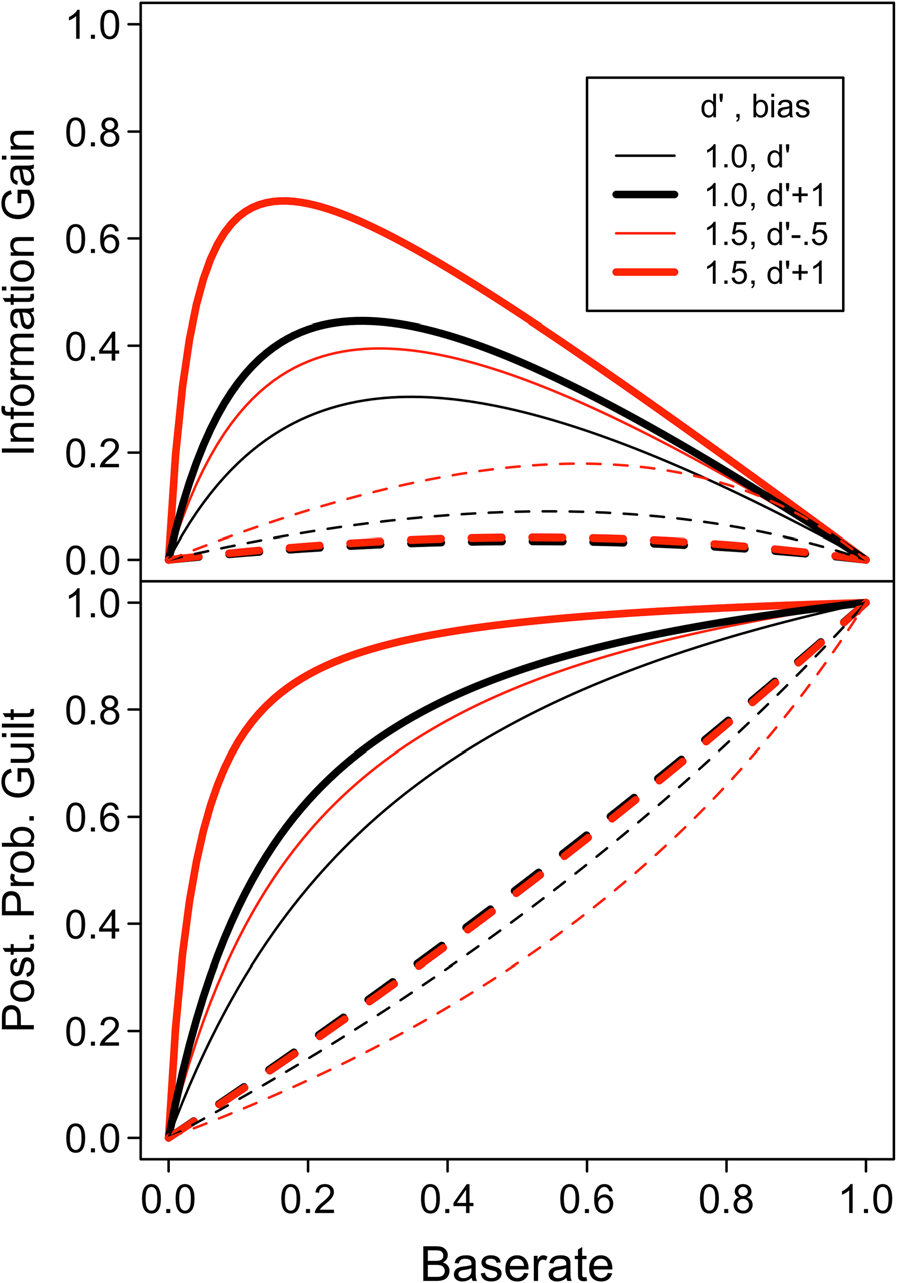
Bayesian measures of eyewitness identification decisions. *Lower panel*: Posterior probability of guilt after suspect identifications (*solid curves*) and filler identification or lineup rejections (*dashed curves*) for two levels of true discrimination and various response criteria. *Upper panel*: Information gain (posterior probability of guilt − base rate) for the same data. Bias is defined by the location of the decision criterion in standard deviation units (e.g., *d‘* + 1 indicates a criterion located 1 standard deviation above the mean of the guilty suspect distribution mean)

To demonstrate the problem of interpreting information gain curves and posterior probabilities, we generated hit and false alarm rate pairs under the assumption of two different true discrimination levels (*d′* = 1.0 or 1.5), each with two different response biases or confidence levels that were selected for illustrative purposes. We then used those data to calculate the posterior probability of guilt and the information gain curve for each combination of discrimination and bias; these are displayed in Fig. 4. It is apparent in Fig. 4 that both greater discrimination and a more conservative response bias (or higher confidence level) result in greater information gain from a positive identification. The condition with lower true discrimination of guilty from innocent suspects may nonetheless yield greater information gain from a positive identification *if* the response bias is conservative. This effect may explain the information gain curves derived from sequential and simultaneous lineups (Wells et al., 2015a).

Information gain curves and posterior probabilities of guilt reflect a combination of the probability that a guilty suspect is presented to the witness (base rate of TP lineups), the witness’s willingness to identify someone (response bias or confidence), and the true discrimination potential provided by the identification procedure. As we have seen, ROCs provide the best way of independently assessing response bias and witness discrimination of guilty from innocent suspects.

## ROCs provide constraints on theory development

In this final section, we suggest some directions for the field in which ROCs might play a particularly important role. As Lampinen (2016) also noted, the field needs a process model of eyewitness identifications. Clark’s (2003) WITNESS model provides a good starting point that is grounded in the broader literature on memory modeling, and we hope that additional work with WITNESS and alternative models will be forthcoming. However, the absence of an influential and broadly applied process model is not an argument against the use of ROCs. Indeed, we would argue that ROC data offer essential constraints on the development of any process model of eyewitness identifications, just as they have constrained development of process models of recognition memory more generally (e.g., Ratcliff & McKoon, 1991; Shiffrin & Steyvers, 1997).

To demonstrate some of the potential of ROCs for both constraining theory development and guiding decisions about experimental paths to pursue or to avoid, we simulated a few different decision strategies that have been proposed in the literature. The simulated data were then used to generate the ROC that would result if that hypothetical decision process were used; in principle, these theoretical ROCs can be compared with empirical data. This approach can be particularly useful for eliminating potential decisional processes from further consideration if, for example, the theoretical ROC fails to correspond to empirically observed ROCs.

In our first simulation, we considered two decision rules that have been proposed for identification decisions in a simultaneous lineup procedure, namely the absolute strength rule, in which the strongest above-criterion photograph is selected (Clark, 2003; Lampinen, 2016), and a relative strength rule, in which an identification is made only if the strongest above-criterion photograph exceeds the next-best photograph by some amount (Clark, 2003; Davey: Absolute and relative decision rules in eyewitness identification, unpublished dissertation). If the two strongest items both exceed the criterion but their difference is too small to allow identification, then the witness could either guess (identifying one of them at random) or reject the lineup. We considered both variants.

**Fig. 5:**
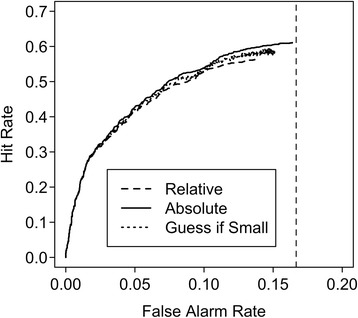
Simulated receiver operating characteristic curves using three possible decision rules in a six-person simultaneous lineup with true *d′* = 1.5. Absolute rule: The strongest item above criterion is selected. Relative rule: The strongest above-criterion item is selected if it exceeds the next best item by a minimum amount (here, 0.15 *d′* units); otherwise, the lineup is rejected. Guess if small rule: Same as the relative rule, except that the witness is assumed to guess if the difference between the two strongest items is too small (<0.15 *d′* units)

For these simulations, we used the same sampling process as we described previously. For TP lineups, one value was sampled from a normal distribution with a mean of *d′* = 1.5 and a standard deviation of 1, and five values were sampled from a distribution with a mean of 0 and standard deviation of 1; for TA lineups, six values were randomly sampled from an *N*(0,1) distribution. This sampling process was repeated 10,000 times. The criterion location was varied in small steps over a wide range; for the relative decision rules, the minimum difference in strengths was arbitrarily set to 0.15, one-tenth the true discrimination value. The results, shown in Fig. 5, suggest that empirically distinguishing these three possible decision rules is likely to be difficult or impossible: The ROCs are essentially identical, except that the two relative decision rules result in responses that appear ever so slightly more conservative than those based on the absolute rule. This effect is a consequence of the decision rule itself: The same evidence values and response criteria were used in all three simulations.

**Fig. 6:**
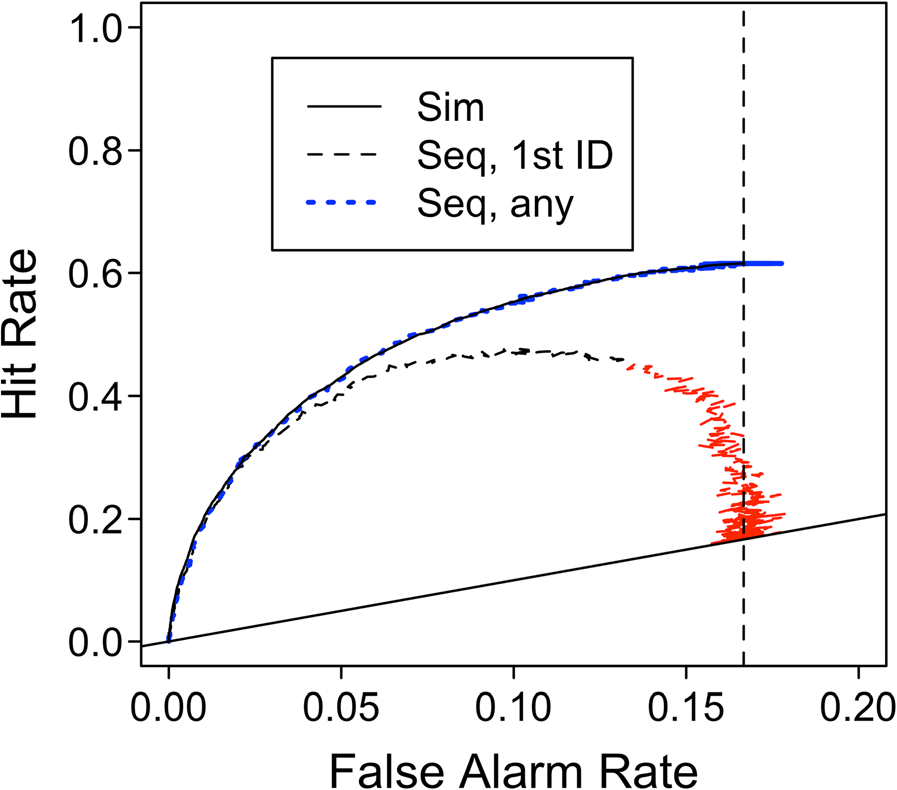
Simulated receiver operating characteristic curves for sequential and simultaneous (Sim) six-person lineups assuming true *d′* = 1.5. An absolute decision rule was used for the simultaneous lineup: The strongest above-criterion item was selected. Two versions of the sequential task were simulated, one in which the first tested item to exceed criterion was identified, at which point the trial ended (Seq, 1st ID), and the other in which the trial continued after an initial identification; subsequent identifications of stronger items were allowed (Seq, any)

In our second simulation, we compared a six-person simultaneous lineup task with a sequential identification procedure using the same sampled evidence values. Simulation of the simultaneous task was identical to that used to generate Fig. 1: An absolute decision rule was used. In the sequential task, we assumed that our “witnesses” were asked to make a yes-or-no identification decision for each individual in the six-person lineup using a set of simple rules: Any strength above criterion resulted in a positive identification, a positive identification ended the procedure, and only a single “lap” through the photographs was allowed. The presentation order of the photos in the sequential simulation was randomized for every simulated lineup, and the response criterion was varied across a wide range of possible values to allow us to map the entire ROC. The results are shown with the black functions in Fig. 6 for *d′* = 1.5.

Although the simulated simultaneous lineup ROC looks quite similar to those observed empirically (e.g., Mickes et al., 2012), the simulated “first-identification-only” sequential lineup ROC has a surprising nonmonotonic form. As the criterion becomes more liberal, both the hit and false alarm rates increase, but only to a point: Once the criterion is too liberal, positive identification of filler items in the TP lineup increases, which ends the trial without allowing a correct identification to occur. Because there are five filler items in the TP lineup, there is a high probability that at least one of them will appear earlier in the sequence than the guilty suspect does, resulting in an ironically reduced hit rate at more liberal criterion locations. (This part of the ROC is shown in *red*; in this region, the criterion location is placed no more conservatively than *d′*/2.) In the limit, if the criterion is set so low that any photo is given a positive response, then the hit rate must simply equal the probability that the guilty suspect appears first in the sequence, or one in six for our simulated six-person lineups. Importantly, the same memory strengths were used on both simulated lineup tasks, and thus true discrimination of guilty from innocent suspects is equated for these ROCs. The curves diverge only because of differences in the specific decision processes we simulated.

How do we interpret the results of our second simulation? Although the resulting ROCs appear to imply that the AUC will be lower for sequential than for simultaneous lineups, that conclusion holds only if witnesses make decisions exactly as we simulated in each task—fixed criterion location for each trial, no changes in criterion placement as additional photographs are shown, absolute decision on each photo, completely randomized photograph order, *and* the same criterion location and true *d′* value for every witness. Of course, experimental conditions and “real-world” identification procedures will violate these assumptions, if for no other reason than individual witnesses surely have different memory abilities and response biases (e.g., Kantner & Lindsay, 2012).

It is also common for sequential lineup identification tasks to allow both experimental and actual witnesses to see the sequence of photographs more than once (e.g., Horry, Brewer, Weber, & Palmer, 2015; Wells, 2014), and changing that aspect of the simulation changes the resulting theoretical ROC. The *blue dashed function* in Fig. 6 shows the ROC that results derived from a sequential identification task in which the witness is allowed to make more than one identification; the identified photograph that has the strongest match to memory is selected as the final choice. This sequential lineup simulation, in which any identification of the guilty suspect counts as long as it is stronger than any previously identified filler, yields an ROC that looks exactly like the simultaneous lineup ROC. Thus, we conclude that the detailed comparison of sequential and simultaneous lineup ROCs depends heavily on exactly the methodology used in administering the lineups and conducting the simulations.

We hope that these examples demonstrate the power of simulation work in the context of eyewitness ROCs: Assumptions about memory and decision processes can be made explicit and tested against data. Precisely this sort of simulation and data-fitting process has been instrumental in development of theory in the broader literature on memory (e.g., Clark & Gronlund, 1996; Shiffrin & Steyvers, 1997), and we have every reason to believe that it offers the same advantages in the subfield of eyewitness identifications. Wixted and Mickes (2014) provided a first step in that direction; we hope that others will follow their lead.

## Conclusions

The resolution of the debate about whether diagnosticity or AUC provides a better measure of witnesses’ ability to discriminate between guilty and innocent suspects is clear. The empirical data are inconsistent with the measurement assumptions of diagnosticity, and consistent with those offered by signal detection theory’s ROCs. This fact alone is sufficient to eliminate diagnosticity from consideration as a measure of witness discrimination accuracy: Diagnosticity and response bias are completely confounded.

The criticisms of ROC-based interpretations of eyewitness identifications are in many cases simply wrong: The area under the curve really does measure discrimination accuracy (*d′*) and does not provide information that is redundant with diagnosticity. ROCs do not “compare hit rates after equating false alarm rates” (Lampinen, 2016, p. 32). Thus, we believe the ROC-based conclusion that showup identifications yield lower-accuracy decisions than lineup identifications is well-founded (Gronlund et al., 2012; Mickes, 2015; Wetmore et al., 2015), as are the conclusions that there is certainly no evidence for a sequential lineup advantage and that there may actually be a simultaneous advantage (e.g., Mickes et al., 2012). Other ROC criticisms are simply irrelevant: Filler siphoning, cost-benefit analyses, and the base rate of TP lineups do not influence the AUC that summarizes the accuracy of suspect identifications. Continued argument along these lines will serve only to hinder research progress.

ROCs *eliminate the need* to worry about possibly differing biases across test conditions because, by definition, they have the same discrimination accuracy (*d′*) at all possible response biases. This clear advantage of ROC analyses has fueled theoretical advances in other domains, including recognition memory (Dougal & Rotello, 2007; Shiffrin & Steyvers, 1997), metamemory (Benjamin & Diaz, 2008; Masson & Rotello, 2009), reasoning (e.g., Dube et al., 2010; Heit & Rotello, 2014), medical diagnostics (e.g., Metz, 1986), veterinary medicine (e.g., Gardiner & Greiner, 2006), weather forecasting (e.g., Mason & Graham, 2002), and machine learning (Furnkranz & Flach, 2005). There is every reason to believe that ROC analyses will be just as important to understanding eyewitness identification decisions.

## Footnotes

1 When discussing the diagnosticity ratio, Wells and Lindsay (1980, p. 778) stated that “Functionally, an identification of a foil is treated as a nonidentification as is a witness’s choice of making no identification from the lineup (i.e., exercising a none-of-the-above option).”

2 As we will see in the discussion of Fig. 1, these reductions in the hit and false alarm rates due to filler siphoning occur even if witnesses’ decision criteria and memory evidence are identical.

3
*d′* is the distance between the means of the target and lure distributions, measured in units of their shared standard deviation. If the distributions have the same variance, then *d′* reflects subjects’ discrimination of targets and lures. If the distributions do not have equal variances, then *d′* is confounded with response bias and should not be used.

4 Our conclusions are restricted to the specific model that we, and Lampinen (2016), simulated. Different assumptions could result in different ROCs, a point we revisit in more detail when we consider how ROCs could constrain theoretical development in the eyewitness domain.

5 To be sure that our conclusions did not depend on the particular *d′* values we selected, we reran these pAUC simulations for 16 levels of *d′* (from 0.25 to 4.0 in increments of 0.25). For *d′* < 3.0 (a high limit for eyewitness identification accuracy), even these small increments in *d′* resulted in observable differences in pAUC for both lineups and showups. For *d′* ≥ 3.0, the confidence intervals grew quite wide. The comparisons of pAUC for showup and lineup identifications at a given *d′* were similar to those shown in Fig. 2.

6 The proof is taught in elementary statistics courses: The distribution of the difference between two independent random variables, *x* ~ *N*(μ_x_, σ_x_) and *y* ~ *N*(μ_y_, σ_y_), is (*x* − *y*) ~ *N*(μ_x_ − μ_y_, √[σ^2^_x_ + σ^2^_y_]).
